# GWASeq: targeted re-sequencing follow up to GWAS

**DOI:** 10.1186/s12864-016-2459-y

**Published:** 2016-03-03

**Authors:** Matthew P. Salomon, Wai Lok Sibon Li, Christopher K. Edlund, John Morrison, Barbara K. Fortini, Aung Ko Win, David V. Conti, Duncan C. Thomas, David Duggan, Daniel D. Buchanan, Mark A. Jenkins, John L. Hopper, Steven Gallinger, Loïc Le Marchand, Polly A. Newcomb, Graham Casey, Paul Marjoram

**Affiliations:** Department of Preventive Medicine, Keck School of Medicine of the University of Southern California, Los Angeles, CA USA; Department of Molecular Oncology, John Wayne Cancer Institute at Providence Saint John’s Health Center, Santa Monica, CA USA; Centre for Epidemiology and Biostatistics, Melbourne School of Population and Global Health, The University of Melbourne, Parkville, Melbourne, VIC Australia; Translational Genomics Research Institute, Phoenix, AZ USA; Oncogenomics Group, Genetic Epidemiology Laboratory, Department of Pathology, The University of Melbourne, Parkville, Melbourne, VIC Australia; Samuel Lunenfeld Research Institute, Mount Sinai Hospital, Toronto, ON Canada; University of Hawaii Cancer Center, Honolulu, HI USA; Public Health Sciences Division, Fred Hutchinson Cancer Research Center, Seattle, WA USA

## Abstract

**Background:**

For the last decade the conceptual framework of the Genome-Wide Association Study (GWAS) has dominated the investigation of human disease and other complex traits. While GWAS have been successful in identifying a large number of variants associated with various phenotypes, the overall amount of heritability explained by these variants remains small. This raises the question of how best to follow up on a GWAS, localize causal variants accounting for GWAS hits, and as a consequence explain more of the so-called “missing” heritability. Advances in high throughput sequencing technologies now allow for the efficient and cost-effective collection of vast amounts of fine-scale genomic data to complement GWAS.

**Results:**

We investigate these issues using a colon cancer dataset. After QC, our data consisted of 1993 cases, 899 controls. Using marginal tests of associations, we identify 10 variants distributed among six targeted regions that are significantly associated with colorectal cancer, with eight of the variants being novel to this study. Additionally, we perform so-called ‘SNP-set’ tests of association and identify two sets of variants that implicate both common and rare variants in the etiology of colorectal cancer.

**Conclusions:**

Here we present a large-scale targeted re-sequencing resource focusing on genomic regions implicated in colorectal cancer susceptibility previously identified in several GWAS, which aims to 1) provide fine-scale targeted sequencing data for fine-mapping and 2) provide data resources to address methodological questions regarding the design of sequencing-based follow-up studies to GWAS. Additionally, we show that this strategy successfully identifies novel variants associated with colorectal cancer susceptibility and can implicate both common and rare variants.

**Electronic supplementary material:**

The online version of this article (doi:10.1186/s12864-016-2459-y) contains supplementary material, which is available to authorized users.

## Background

We live in the era of the Genome-wide Association Study [GWAS]. Large numbers of samples have been collected and genotyped in a bid to associate Single Nucleotide Polymorphisms [SNPs] with phenotypic variation. In the context of human disease, the design of such studies and, in particular, the so-called SNP-chip technology that underpins them, has aimed to exploit the *common disease, common variant* hypothesis (e.g.), [1, 2]. This assumes that common diseases will frequently be associated with common (>1–5 % frequency) variants.

There is now a long history of GWAS studies, and large numbers of variants have been found to be associated with disease [3]. However, such studies do not come without financial cost, and there has also been a lively discussions regarding whether such a track record should be regarded as a success or failure [4, 5]. Our purpose here is not to add to that discussion, but rather to focus on what will often be a frequent ‘next step’ in such studies.

While it is undeniable that GWAS has uncovered large numbers of variants that are associated with disease, it has also become clear that, while these variants do appear to be associated with disease, they can only explain a fraction of the phenotypic variation that is observed. Unfortunately, this fraction is general very low (e.g.), [6]; but see, also, [7]. Such demonstrations of *missing heritability* have lead to some skepticism about the common disease, common variant hypothesis [e.g., 5].

There are many possible explanations for missing heritability (see, e.g.), [4, 5] for discussions. These include rare variants, complex genetic architectures, structural variation such as copy number variation, the joint effects of large numbers of variants each of small effect marginally, and so-called phantom heritability (e.g.), [5, 7, 8]. In this paper we focus on the first of those hypotheses: the discovery of nearby, and possibly rare variants that drive GWAS signals.

Because of their focus on common variants, SNP-chip platforms were not well placed to discover associations between disease and rare genetic variants. Theoretically, discovery through so-called synthetic associations with common variants is possible [9], although we note that there is some discussion regarding whether such phenomena are likely to explain most GWAS signals (e.g.), [10, 11]. However, given this possibility, combined with the recognition that an initial GWAS may well be finding SNPs that are not causative in themselves, but are instead linked with nearby causative polymorphisms, there has been a move towards following-up GWAS studies by sequencing studies (e.g.), [12]. Here, the hope is that a signal of association that has been found in GWAS can be refined, and strengthened, by sequencing the region of the genome that surrounds the *focal SNP* (the SNP that was observed to have a small p-value in the original GWAS). Alternative strategies, that are not the subject of the present paper, include whole-genome or whole-exome sequencing (e.g.) [13].

However, before such a sequencing study can be conducted, several design questions must be resolved (where to sequence, at what depth, etc.). With this in mind, NIH formed the GWASeq consortium, in which multiple groups were funded to conduct sequence-based follow-up to GWAS, and thereby create a pool of publically-available data that could both a) provide the potential for refinement of GWAS signal for the phenotypes of interest, and b) provide a publically-available resource that the wider community might use to help guide their approach to such design questions for their own studies. The study we describe in this paper is one member of the GWASeq consortium. As such, the data are in the process of being made publically available through dbGaP, the NCBI’s repository for data that attempt to relate genotype to phenotype (http://www.ncbi.nlm.nih.gov/gap).

It should be noted, of course, that a large number of studies outside the GWASeq consortium are also attempting to follow-up GWAS hits using NGS technology, and examples are beginning to appear. An early example of this is Nejentsev et al. [14], in which the authors sequenced exons and splice-sites for ten candidate genes that contained previously associated common SNPs for type-1 diabetes in order to identify rare functional variants. Likewise, there are also a growing number of exome-sequencing studies, e.g. Liu, et al. [13], that focus on testing for rare functional variants.

Our study focuses on colorectal cancer. Colorectal cancer is the fourth-most common cancer and the second-most common cause of cancer death in the United States, with approximately 148,810 new cases and 49,960 deaths estimated in 2008 [15]. There is known to be a strong genetic component to CRC risk, and individuals with a family history of colorectal cancer are at increased risk of the disease. For example, having a first-degree relative with CRC roughly doubles the risk, [16]. Further evidence of heritability is seen in twin studies. For example, in a large twin study, up to 35 % (95 % CI: 10 % to 48 %) of CRC risk could be explained by inherited factors [17]. GWAS hits have been found in a number of regions: 8q23.3 (rs16892766), 8q24 (rs6983267, rs7014346, rs10505477) [18–22], 9p24 (rs719725) [19, 20], 8q23.3 (rs16892766, *EIF3H*) and 10p14 (rs10795668) [18], 11q23 (rs3802824) [22], 12q13.13 (rs7136702), 14q22.2 (rs4444235), 15q13.3 (rs4779584) [23], 18q21 (rs4939827, *SMAD7*) [22, 24], and 20q13.33 (rs4925386). It is these regions that form the basis for follow-up in our experimental design.

Our data consist of samples from the Colon Cancer Family Registry [CCFR, http://www.coloncfr.org] [25]. The CCFR includes data and biospecimens from over 42,500 total subjects (~15,000 probands and 27,500 selected unaffected and affected relatives and unrelated controls). The consortium consists of six research institutions. In the present study we include germ-line samples from 5 of those centers (Table 1). A total of 4,052 samples were sequenced. A sub-set of these samples consisted of pedigree-based samples (~1,000 samples) – these do not form part of the analysis described in this paper. After a variety of Quality Control checks (see Methods), we conducted our analyses using 1993 cases and 899 controls.

**Table 1:** Sample information for all samples sequenced in this study

CCFR center	Num. samples	Population based	Pedigree	Buccal
Australia	1,664	1,155	509	2
USC	370	88	282	266
Seattle	910	778	132	0
Mt. Sinai	1, 007	924	83	0
Hawaii	101	101	0	0
Totals	4,052	3,046	1,006	268

Both population based and pedigree based samples were included in the sequencing. The majority of samples were sequenced from genomic DNA extracted from stored blood, with a sub-set of samples that were sequenced from stored buccal swabs

## Results

### Sequencing

**Fig. 1:**
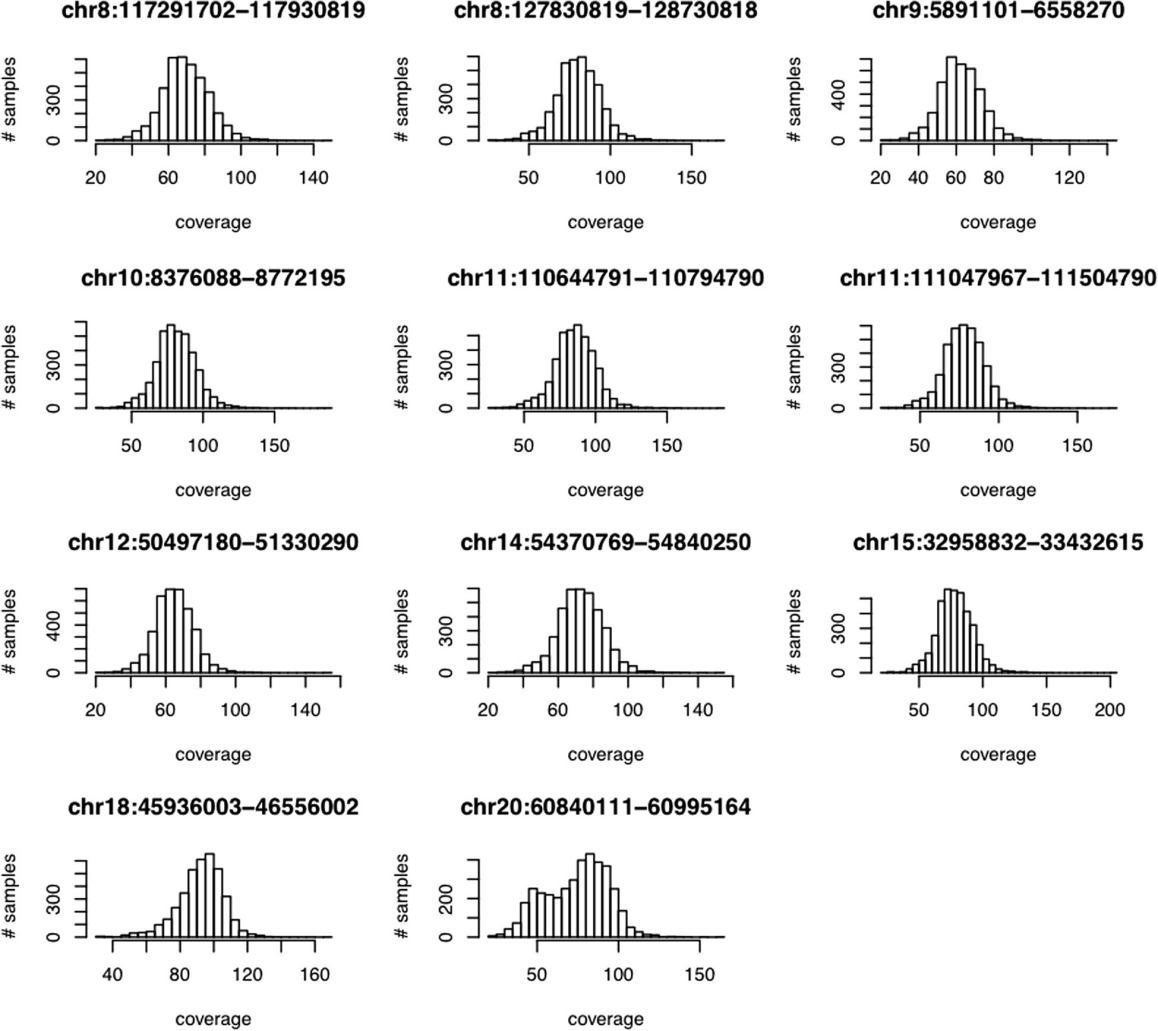
Mean coverage across all 11 targeted regions. The x-axis is the mean coverage for each sample. The y-axis is the number of samples at a given coverage. See text for discussion of the bimodal distribution of coverage across chr20:60840111–60995164

Our samples were sequenced at the Baylor College of Medicine [BCM] sequencing center. In all, 4,052 samples were successfully sequenced and passed all of the BCM’s internal quality controls. An overview of the sequencing results is presented in Table 2. For each sample, approximately 5.8 MB of the genome was sequenced to an average depth of 76X (Fig. 1). To explore how well each targeted region was covered by the sequencing we calculated the breadth of coverage across each targeted region. On average, approximately 80 % of the targeted regions were covered at 30X or greater (Table 2). The distribution of coverage was similar among all the targeted regions except for the 20q13.33 region. We originally suspected that the differences in the observed coverage for this region might be due to structural variation affecting mapping, but after closer inspection we did not detect any large-scale structural variation in this region. Rather, it is the case that a subset of our samples appears to have lower coverage over all regions, and, for reasons that are unclear, this effect appears to be magnified for the 20q13.33 region. There is no evidence of differential coverage rates between cases and controls (see Additional file 1).

**Table 2:** Summary of 11 genomic regions sequenced

SNP	Band	Region sequenced	Total sequenced (bp)	Mean coverage	% of target with > = 30X	Uncorrected *p*-value (0 PCs)	Uncorrected *p*-value (2 PCs)
rs16892766	8q23.3	8:117,291,701–117,930,819	639,118	69.58	77.25	0.04119	0.03253
rs10505477	8q24	8:127,830,818–128,730,818	900,000	80.17	82.35	0.02315	0.01395
rs719725	9p24	9:5,891,100–6,558,270	667,170	62.40	66.81	0.05862	0.05821
rs10795668	10p14	10:8,376,087–8,772,195	396,108	81.17	81.86	0.002037	0.00187
rs3802842	11q23	11:110,644,790–110,794,790	150,000	85.54	86.64	0.002948	0.002393
rs3802842	11q23	11:111,047,966–111,504,790	456,824	85.54	86.64	-	-
rs7136702	12q13.13	12:50,497,179–51,330,290	833,111	64.97	80.70	0.003239	0.006124
rs4444235	14q22.2	14:54,370,768–54,840,250	469,482	73.01	78.49	0.5992	0.746
rs4779584	15q13.3	15:32,958,831–33,432,615	473,784	78.31	82.85	0.08478	0.4264
rs4939827	18q21	18:45,936,002–46,556,002	620,000	92.27	86.52	0.02014	0.008508
rs4925386	20q13.33	20:60,840,110–60,995,164	155,054	74.18	72.77	0.1239	0.0981
		Totals	5,760,651	76.16	79.62		

The first column indicates the focal GWAS SNP that the region was designed around. Sequencing coverage for each region was calculated as the mean coverage across the entire targeted region and as the breadth of coverage. The breadth of coverage is defined as the number of bases per targeted region that are coverage at > = 30X coverage

### Variant calls

**Fig. 2:**
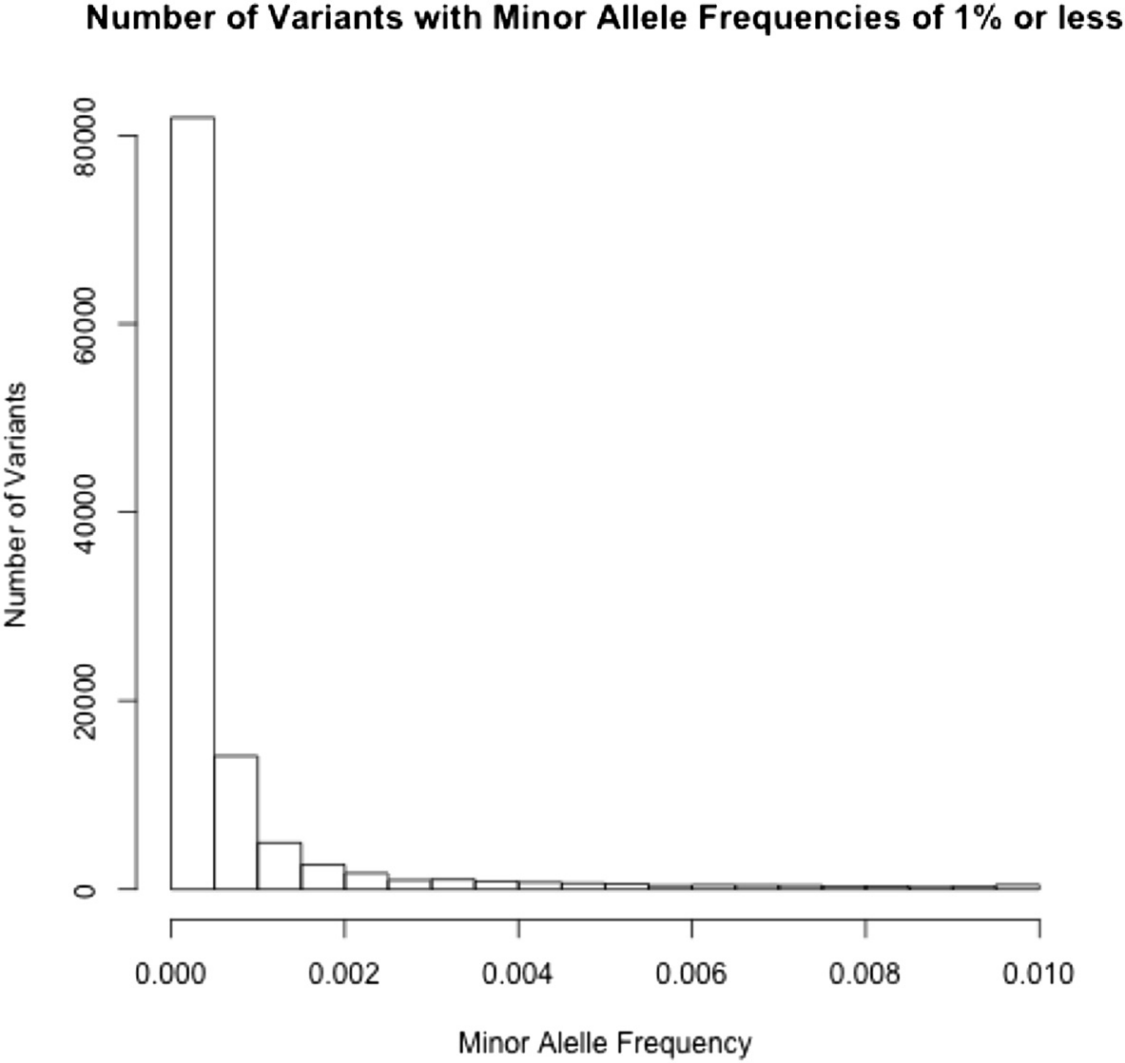
Distribution of allele s with a MAF of less than 0.01 for the 2,838 population based samples

We identified a total of 192,991 polymorphic sites in the 4,052 samples. Of these sites, 139,394 were found to be novel (~72 %) and had not been previously identified in either dbSNP (version 137) or as part of the 1000 Genomes project (The 1000 Genomes Project Consortium, 2010). After filtering (see Methods), we retained 158,774 (~82 %) of the originally identified polymorphic sites. For the non-novel variants, we compared ‘consistency' of our variant calls with those of the 1000 Genomes project. Specifically, we checked whether the variant allele observed in our data was the same as that seen in the 1000 Genomes data. The consistency between our raw and filtered variant call sets compared with 1000 Genomes data was ~97.49 % for both call sets. This similarity in consistency between our raw and filtered call sets is reflective of the fact that both call sets accurately detected the more “common” variant sites from the 1000 Genomes data set. The vast majority (>89 %) of variants identified in this study have a MAF < = 0.01 (Fig. 2).

### Sample QC

Overall call rates and concordance between genotypes identified in the sequence data as compared to genotypes called from previously collected SNP array data (where they exist) was high. We identified 33 samples where the concordance between the sequence-based genotype calls and one or more array-based genotype calls was low and therefore we removed those samples from the analysis. Given the high concordance between our sequence-based genotype calls and the array-based genotype calls we believe that the number of mis-identified or mis-labeled samples in our final data set is negligible.

The logistical constraints of this study, in which data was shipped from a variety of centers at a variety of times, and sequencing was necessarily performed in batches at a third-party center, meant that we were not able to explicitly design the study to guard against batch or center effects during sequencing. However, we carefully examined the sequence data for batch and center effects and found none. We saw no evidence of any clustering by center in PC plots, nor variation in coverage by center. We also examined SNP density, $\pi$, for each center and means were very similar (ranging from 0.00100 to 0.00106).

**Fig. 3:**
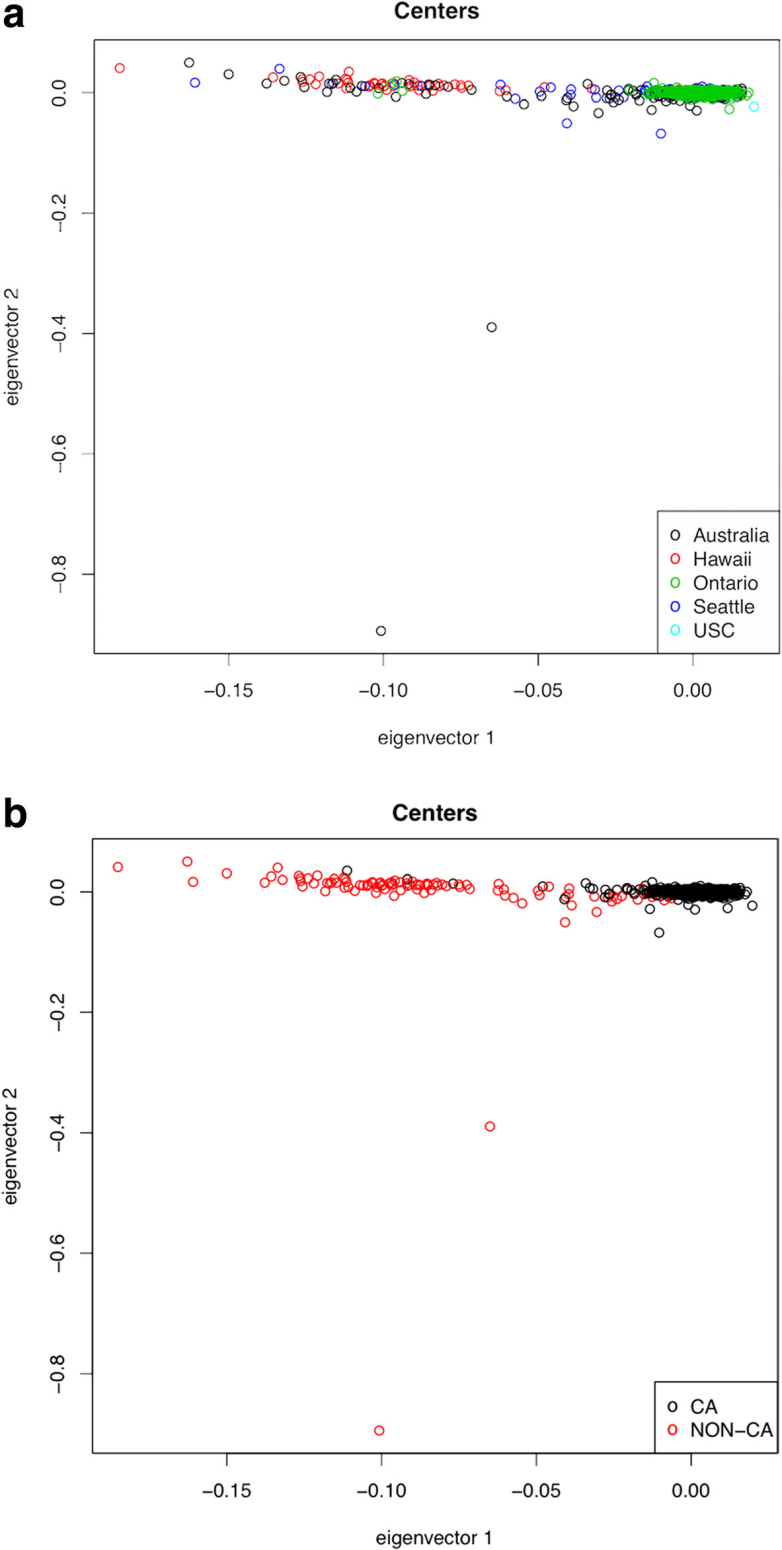
**a** and **b**. PCA analysis of the population based samples. **a**. PCAs colored by CCFR center. **b**. PCAs colored by race with all non-Caucasian individuals colored in *red* and Caucasian samples colored in *black*

In addition, we performed a Principal Component analysis to identify other apparent sample outliers. We removed 2 samples that were revealed as outliers by plotting data on PC axes (Fig. 3, see Methods for more details) and then recalculated PC axes based on the remaining samples.

### Association tests

In this paper we focus upon testing for association in the population-based samples that passed the preceding QC checks (1993 cases, 899 controls). Analysis of the family-based data is ongoing and will be presented in a separate paper.

### Population structure and candidate region studies

It is traditional to control for population structure in a GWAS context. In order to do this, global genome-wide structure and relatedness between samples is evaluated using a genome-wide set of (roughly unlinked) markers. In the present study, which amounts to a study of 11 candidate regions, this is impossible. While we chose to calculate PCs as part of the QC process, to uncover obvious outliers, it is entirely unclear that such PCs will reliably capture genome-wide patterns of relatedness. Neither do we have ‘SNP-chip’ data for every sample in our data. We have a total of just ~ 5.8 MB of data per sample, divided into 11 short (~500 KB) regions. As such, we believe it is likely that PCs calculated form this data may capture local, rather than global structure. Indeed we see no correspondence between PCs calculated on the samples retained for the association analysis and ethnicity or center of collection. We also note that the first PC explains <4 % of the variation in the sample. This lack of structure is consistent with the vast majority (>97 %) of our samples being comprised of Caucasian individuals, and is further consistent with the structure that is observed in earlier GWAS analyses using CCFR samples and common SNPs.

For this reason, while for comparison’s sake, we present results for analyses that include both 0 and 2 PCs, we propose to focus on the results for the analysis containing 0 PCs. We return to this point in the next section.

### (Non-rare) variant associations

As a reflection of the reduction of power to detect associations as variant MAF decreases, we focus our marginal tests of association on variants with MAF > 0.005. This results in us testing a total of 23,855 variant positions. We identified 10 variants (or 9 in the analysis that includes 2 PCs) distributed among six of the targeted regions at a FDR significance level of 0.01 (Table 3). Eight of the 10 variants were novel to this study. All of these variants were located in non-coding regions of the genome. Of the 10 variants, 7 were located in the intronic regions of the genes *KIAA2026*, *CERS5*, *TMPRSS12*, *FMN1*, *CTIF and LOXHD1*. The remaining 3 variants were located in the intergenic regions between genes LINC00708 and LINC00709, *BMP4* and *CDKN3*, and *SGG5* and *GREM1* (see Table 3 for distances). Detailed regional plots for the above significant associations and all regions tested are presented in Additional file 2.

**Table 3:** Most significantly associated SNPs identified in the PLINK analysis

Chr: Position	rs ID number	Feature	Base change	MAF: Cases (Controls)	Gene (distance to nearest genes)	Uncorrected p-value (0PCs)	FDR corrected (0PCs)	Uncorrected (2PCs)	FDR corrected (2PCs)
9: 5,980,030	Novel	intronic	A to G	0.048(0.065)	KIAA2026	5.323e–008	0.0002117	1.587e–007	0.0005407
10: 8,542,529	Novel	Intergenic	T to G	0.016(0.028)	LINC00708(232261), LINC00709(775047)	3.576e–007	0.0009478	2.461e–007	0.000734
12: 50,554,103	Novel	intronic	A to G	0.026(0.033)	CERS5	2.926e–007	0.0008725	6.119e–007	0.001622
12: 51,243,510	Novel	intronic	A to T	0.049(0.057)	TMPRSS12	7.362e–008	0.0002509	1.104e–007	0.0004388
14: 54,603,486	rs116055771	Intergenic	A to T	0.013(0.022)	BMP4(179,932), CDKN3(260,187)	4.085e–011	3.248e–007	2.014e–010	1.602e–006
15: 33,008,360	Novel	Intergenic	A to C	0.034(0.054)	SCG5(19,062), GREM1(1,845)	6.384e–009	3.046e–005	2.652e–008	0.0001265
15: 33,345,877	Novel	intronic	A to C	0.004(0.011)	FMN1	2.048e–011	2.443e–007	6.591e–011	7.861e–007
18: 46,119,756	Novel	intronic	T to C	0.010(0.021)	CTIF	2.128e–009	1.736e–006	5.41e–010	3.226e–006
18: 46,119,757	rs76590328	intronic	C to T	0.031(0.054)	CTIF	6.103e–013	1.456e–008	1.98e–012	4.724e–008
18: 46,503,254	Novel	intronic	A to G	0.031(0.057)	LOXHD1	2.722e–006	0.006494	4.838e–006	0.01154

**Fig. 4:**
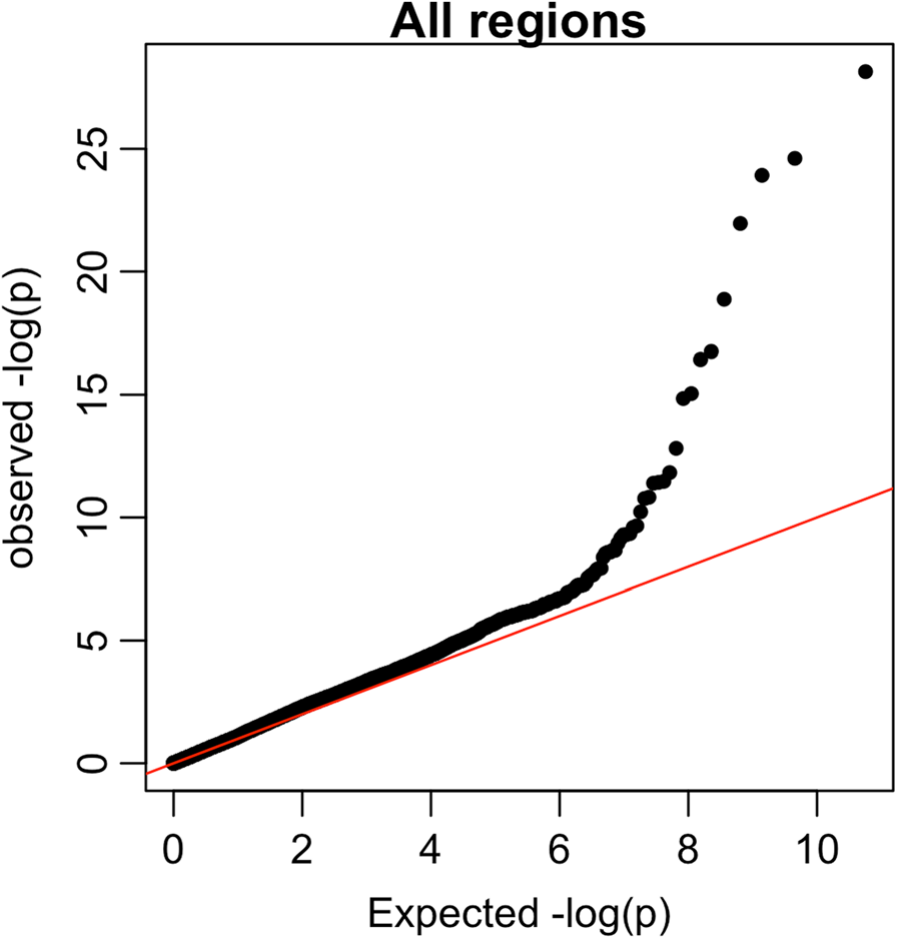
QQ-plot for marginal associations between common polymorphism and cancer status across all sequenced regions. X-axis shows expected –log(p-value); y-axis shows observed –log(p-value)

We note that we see no evidence of overall inflation of p-value across our regions, despite our choice not to include PCs, or to include just 2 PCs, in the association test (Fig. 4, see Additional file 3 for a breakdown of this plot by region). Rather we see p-value that are distributed as expected under the null, with the exception of an excess of small p-value, which is what one would hope to see in a study such as ours in which we are following–up on putative hits from earlier studies (albeit, in general, from samples not included in the present study).

It is of particular interest to examine the strength of association found with each of the ‘focal’ SNPs around which the 11 regions we defined. This is recorded in the final two columns of Table 2. (Here, we report uncorrected p-value, as if one were conducting a validation study of that SNP alone). We note that, with the exception of rs4444235 and rs4779584 we see a strong tendency for these tests of association to return small p-value. This is, at the very least, encouraging regarding the veracity of those original signals.

### Rare variant associations

To test for associations with rare variants (MAF ≤0.01) we first annotated the genomic locations of all variants in our call set into either exonic, intronic, intergenic, upstream 1 kb or downstream 1 kb of a known gene, UTRs, or non-coding RNAs categories. Based on these classifications we defined 307 variant sets that were used to test for associations using the SKAT combined test [26]. We identified two variant sets (summarized in Table 4) that showed a significant association after correcting for multiple tests. The two significant variant sets were the 3′ UTR of the gene *C11orf53* (0 PCs p = 0.0486, 2PCs p = 0.0275) and the 5′ UTR region of the gene *ATF1* (0 PCs p = 0.0032, 2 PCs p = 0.0056).

**Table 4:** Composition of the significantly associated SNP sets identified in the SKAT combined analysis

GWAS SNP	Gene	Feature	Position	rs ID number	MAF: Cases (Controls)	PLINK p-value	p-value for SNP set
rs3802842	C11orf53	3′ UTR	11:111,156,836	rs3087967	0.32(0.27)	1.00	Uncorrected 3.17e–004 (1.79e–004)
	C11orf53	3′ UTR	11:111,156,857	Novel	0(5.55e–004)	NA	FDR corrected 0.0486 (0.0275)
	C11orf53	3′ UTR	11:111,156,877	Novel	2.51e–004(0)		
	C11orf53	3′ UTR	11:111,156,937	Novel	0(5.55e–004)	NA	
rs7136702	ATF1	5′ UTR	12:51,157,849	Novel	4.92e–003(7.96e–003)	0.8771	Uncorrected 1.04e–005 (1.83e–005)
	ATF1	5′ UTR	12:51,157,852	Novel	1.04e–003(0)	NA	
	ATF1	5′ UTR	12:51,157,863	rs61926301	0.58(0.61)	1.00	FDR corrected 0.0032 (0.0056)
	ATF1	5′ UTR	12:51,157,886	Novel	2.55e–004(0)	NA	
	ATF1	5′ UTR	12:51,157,960	Novel	2.59e–004(0)	NA	
	ATF1	5′ UTR	12:51,157,996	Novel	2.62e–004(0)		
	ATF1	5′ UTR	12:51,158,010	Novel	2.61e–004(0)	NA	
	ATF1	5′ UTR	12:51,158,027	Novel	0(5.97e–004)	NA	
	ATF1	5′ UTR	12:51,158,045	Novel	7.82e–004(0)	NA	
	ATF1	5′ UTR	12:51,158,047	Novel	2.61e–004(0)	NA	

We then performed SKAT tests for each individual targeted region (see Table 2) separately and conditioned on the original focal GWAS SNP. The single resulting significant variant set is the 5′ UTR of *ATF1* (0 PCs p = 0.0055, 2 PCs p = 0.0052) (Table 5). The *C11orf53* variant set was no longer significant once the focal GWAS SNP was added into the analysis as a covariate.

**Table 5:** Composition of the significantly associated SNP sets identified in the SKAT combined analysis

GWAS SNP	Gene	Feature	Position	rs ID number	MAF: Cases (Controls)	PLINK *p-value*	*p-value* for SNP set
rs7136702	ATF1	5′ UTR	12:51,157,849	Novel	4.92e–003(7.96e–003)	0.8771	Uncorrected 8.08e–005 (7.59e–005)
	ATF1	5′ UTR	12:51,157,852	Novel	1.04e–003(0)	NA	FDR corrected 0.0055 (0.0052)
	ATF1	5′ UTR	12:51,157,863	rs61926301	0.5.(0.61)	1.00	
	ATF1	5′ UTR	12:51,157,886	Novel	2.55e–004(0)	NA	
	ATF1	5′ UTR	12:51,157,960	Novel	2.59e–004(0)	NA	
	ATF1	5′ UTR	12:51,157,996	Novel	2.62e–004(0)		
	ATF1	5′ UTR	12:51,158,010	Novel	2.61e–004(0)	NA	
	ATF1	5′ UTR	12:51,158,027	Novel	0(5.97e–004)	NA	
	ATF1	5′ UTR	12:51,158,045	Novel	7.82e–004(0)	NA	
	ATF1	5′ UTR	12:51,158,047	Novel	2.61e–004(0)	NA	

This analysis was performed on each targeted sequencing region separately and including the focal GWAS SNP as a covariates

## Discussion

Here we present a large-scale data set that we hope will serve as a powerful resource to investigate ways to design a successful strategy for using next-generation sequencing technologies to follow up on GWAS. Given that a GWAS has been preformed and significant associations have identified suspected regions, targeted re-sequencing provides a powerful method to further investigate the fine-scale genomic structure in these regions. However, there are few guidelines as to how such a follow-up study should be performed. For example, design issues include, but are not limited to, the following:

1. Which samples should be sequenced?
2. Which regions should be sequenced?
3. What depth of coverage should be used and how far around the focal SNP should we sequence?
4. To what extent can we rely upon imputation?
5. What designs are more efficient for variant discovery and testing associations?

There are at least two ways one could try to answer design questions such as this. The first is to conduct a large simulation study. Here, data are simulated under a variety of possible disease models, and for a variety of population models and designs for GWAS and subsequent sequencing study. A very large number of variables are at play here, but the advantage of a simulation-based study is that it is possible, at least in principle, to simulate a wide variety of possibilities. The disadvantage, of course, is that the conclusions one draws may or may not be robust to inaccuracies in the underlying simulation model. As was famously noted by George Box, “All models are wrong; some are useful” [27]. The hope is that the conclusions drawn from such an analysis will be useful despite their inevitable (and admitted) inaccuracies. Such studies are extremely computationally intensive, which limits the range of model and design parameters that might be considered, but examples do exist. For example, [28] conducted such an analysis based upon simulating populations of 10,000 genotypes designed to mimic a breast cancer study [29]. They demonstrated that informative sampling based on disease and phenotype status jointly, could improve power, as could incorporating phenotype data from extended pedigree information, in family-based studies.

The second approach to resolving these design questions is data- rather than simulation-based. Here the goal is to collect data in which sequencing has been used to follow-up GWAS hits, and to attempt to draw robust conclusions from those data. Now, as Box might say, the ‘model’ is correct. The data got there however disease data got there (i.e., the model is reality itself). However, the price we pay here is lack of replication - we have a relatively small number of such datasets. Therefore, the challenge will be in drawing robust conclusions from these studies. Our hope is that the data resource described by this paper, and other members of the GWASeq consortium, will help begin to provide those guidelines.

One perspective of the GWASeq consortium is to provide data that are rich enough to enable investigators to assess the effectiveness of alternative designs by subsetting the available data. Given this view, the goal we followed when designing the study was to be conservative in the sense of sequencing at greater depth, for wider regions and more samples, than might otherwise have been the case. This provides maximum scope for assessment of efficiency of alternative designs.

For the 4,052 individuals included in this study we were able to successfully sequence the majority (~80 %) of the intended target region surrounding the GWAS implicated SNP of interest to a sufficient depth to allow us to accurately genotype previously unknown variants in the region. Of the variants we identified, the vast majority (~90 %) of SNPs in our data set are comprised of rare variants with a MAF of less than 0.01. This abundance of rare variants is consistent with the findings of other large-scale sequencing projects that have shown very high levels of genetic diversity present in the human population, driven by recent and rapid population expansion [30].

Of course, a primary interest when collecting data such as these is to determine whether stronger genotype-phenotype associations will be found near the focal SNPs from prior GWAS. Here, we employed two strategies to detect associations and identify variants that confer risk for colorectal cancer susceptibility. The first strategy focused on more “common” variants to determine if any of the higher allele frequency novel variants identified in this data set could be associated with disease susceptibility. This strategy identified 10 new variants, none of which have been previously associated with colorectal cancer. The second strategy we employed was to look at the combined effect of rare and common variants. Here we uncovered associations with distinct variant sets containing both common and rare variants in the 3′UTR of the gene *C11orf53*, and the 5′UTR of the gene *ATF1*.

Previous work to identify the functional risk variants for colorectal cancer in the 11q23.1 region has implicated several genes including *C11orf53* as likely factors in colorectal cancer etiology [31, 32]. Furthermore, Pittman et al. [33] found a variant (rs3087967) in the 3′UTR of *C11orf53* to be in high LD with SNP rs3802842, which was the focal GWAS SNP that our sequencing region was designed around. While we did not detect a significant association with rs3802842, the SKAT combined test did identify three other variants in the same 190 bp region that comprises the 3′UTR of *C11orf53*. The 3′UTR contributes to post transcriptional gene regulation through the regulatory actions of miRNAs. If differences in *C11orf53* expression are involved in colorectal cancer susceptibility, then mutations in the 3′UTR might lead to changes in miRNA binding affinity and thus lead to changes in the expression of that gene. In fact, rs3087967 is directly adjacent to a miR-9 binding sequence (microRNA.org).

Additionally, we detected a significant (*p =* 0.0230, Fisher’s exact test) enrichment of novel variants in cases as compared to controls within the 5′UTR region of the activating transcription factor 1 (*ATF1)* gene, an important cAMP-responsive transcription factor. Two of these novel variant positions lie within an upstream open reading frame (uORF), and three of them lie within the internal ribosome entry site (IRES) [34]. Both uORFs and IRES elements contribute to overall gene expression levels via translation control [35, 36]. In 2012, Huang, et al. [37] showed that expression levels of *ATF1* are positively correlated with survival in colorectal cancer patients.

## Conclusions

This study is likely to be one of a large number of studies that perform targeted sequencing in order to follow-up hits from earlier GWAS. The jury is still out regarding how likely it is that stronger associations will be uncovered by such a strategy, but heritability estimates for many diseases indicate that this is a reasonable hope. In our own study we do find such associations in a number of the regions that we sequenced. However, it is also the case that in a number of the regions no significant signal was found. Regardless, by placing such data in the public domain we hope to enable other groups to better design their own follow-up studies, and thereby increase their own chances of successful discovery.

## Methods

### The study samples

The samples used in this study were taken from the Colon Cancer Family Registry. Informed consent was obtained from all study participants and the study protocol was approved at each center. The overwhelming majority of samples were from DNA extracted from blood samples (~93 %), with the remaining being from buccal cells (~7 %). Each CCFR center individually extracted total genomic DNA and shipped the extracted DNA to the Baylor College of Medicine for sequencing. All study protocols were approved by the USC Health Sciences Institutional Review Board.

### Regions sequenced

A total of 11 genomic regions were selected for targeted re-sequencing (Table 2). For the purpose of this study we define a genomic region as the flanking sequence on either side of a focal GWAS SNP. The amount of flanking sequence surrounding each focal SNP was determined by the local LD structure around the focal SNP, to ensure that the sequenced area extended beyond the LD block containing the focal SNP. The targeted regions were isolated from total genomic DNA using custom designed NimbleGen Sequence Capture Microarrays (following the manufacturer’s protocols). Individual sample libraries were multiplexed together and sequenced on an Illumina HiSeq 2000 at the Baylor College of Medicine. A total of ~5.8 MB of genic and intergenic sequence was collected from each individual sample.

### Sequence mapping

Sequence reads were mapped to the 1000 Genomes (b37) build of the human genome using BWA (version 0.6.2-r126) [38] with default settings. The resulting alignments were further processed using the GATK (version 2.3–9) [39] base quality score recalibration, indel realignment, duplicate removal (picardtools, version 1.84), and read-reduction functions in accordance with the GATK Best Practices recommendations [40].

### Variant calling

Variant detection was performed for polymorphism discovery and genotyping across all 4,052 samples simultaneously using the GATK UnifiedGenotyper (version 2.7–4). We applied an additional mapping quality (MQ) filter of 50 during variant calling to remove false positive variants that result from poor mapping (Additional file 4). The raw variant calls were further refined using the variant quality score recalibration (VQSR) methods according to the GATK Best Practices recommendations [40, 41]. The final variant call set was checked for concordance with both dbSNP (version 137) and 1000 Genomes SNP calls using the GATK AnnotateEval tool and functional annotations were performed using the ANNOVAR annotation pipeline following the authors’ recommendations [42].

### Sample QC

Given the number of samples, repository centers, and individuals involved in the generation of the sequence data, the possibility of some samples becoming mis-labeled is a valid concern. Therefore, for those samples that had existing SNP array data (~2300 samples), we compared the genotype calls from the sequence data to genotype calls from existing SNP array genotyping data generated from the same samples to confirm the identity of as many of the sequenced samples as possible.

Additionally, we tested difference in coverage levels between cases and controls in any of the sequenced regions. Such a difference could induce biases, particularly in the rare variant tests*.* We found no evidence of any statistically significant difference in coverage between cases and controls in any of the regions (see Additional file 1).

### Subset of samples used for associations

A subset of the 4,052 samples that were sequenced consisted of pedigree-based samples (~1,000 samples) - these were removed from the analysis presented in this paper. We also removed 33 samples that were suspected of being mis-labeled (see Sample QC), as well as any samples that lacked full covariate, or phenotypic data. We then conducted a PC analysis, using a set of unlinked variants that covered each of our sequenced regions, using the SNPRelate package in R [43]. This resulted in our removing two samples that represented obvious outliers. PC axes were then recalculated for possible inclusion in association tests. The final data set comprised of 2,892 population-based samples with 1993 cases, 899 controls.

### Association tests

#### Common variants

Marginal tests for association were preformed using the PLINK software package [44]. We used a logistic model and included age at disease diagnoses, sex, CCFR center. Only variants with a genotyping rate > = 95 %, and with MAF > 0.005, were included in the analysis. To correct for multiple testing we applied a false discovery rate (FDR) [45] correction based on the total number of variants tested as implemented in the R function p.adjust.

#### Rare variants

In order to test for associations with rare variants we employed a sequence kernel association test (SKAT) using the SKAT package in R [26]. We employed the combined SKAT test in order to test the combined effect of both common and rare variants [46]. Variant sets comprised of variants annotated in UTRs, exons, introns, within 1 KB of a known gene, and the intergenic sequence between two known genes for a total of 307 variant sets that were included in the analysis. To correct for multiple testing we applied a FRD correction to the raw p-value generated by the combined SKAT test.

## Availability of supporting data

All data used in this article are in the process of being deposited in dbGaP. Readers are encouraged to contact the authors for further details.

## Additional files

Additional file 1: Boxplot of per-sample coverage by region, for each of the sequenced regions, by case–control status. (TIFF 8219 kb) Additional file 2: Regional plots of associations for each targeted region. rs numbers and purple circles indicate the focal GWAS SNP that the region was selected around. Colored circles indicate degree of LD among SNPs. Grey circles indicate novel SNPs that lack LD information based on the 2012 release of the 1000 Genomes data. The rs number at figure top is centered around the location of the focal SNP. (ZIP 1921 kb) Additional file 3: ‘By region’ QQ-plot for marginal associations between common polymorphism and cancer status. X-axes show expected –log(p-value); y-axes shows observed –log(p-value). (TIFF 5627 kb) Additional file 4: Distribution of mapping qualities (MQ) across all samples. Reads with MQ < = 50 were excluded during variant calling to reduce false positive variant calls. (TIFF 2774 kb)
