# Supplementary figures and images for: GWASeq: targeted re-sequencing follow up to GWAS
Source: BMC Genomics. 2016 Mar 3;17:176. doi: 10.1186/s12864-016-2459-y (PMC4776370; doi:10.1186/s12864-016-2459-y)

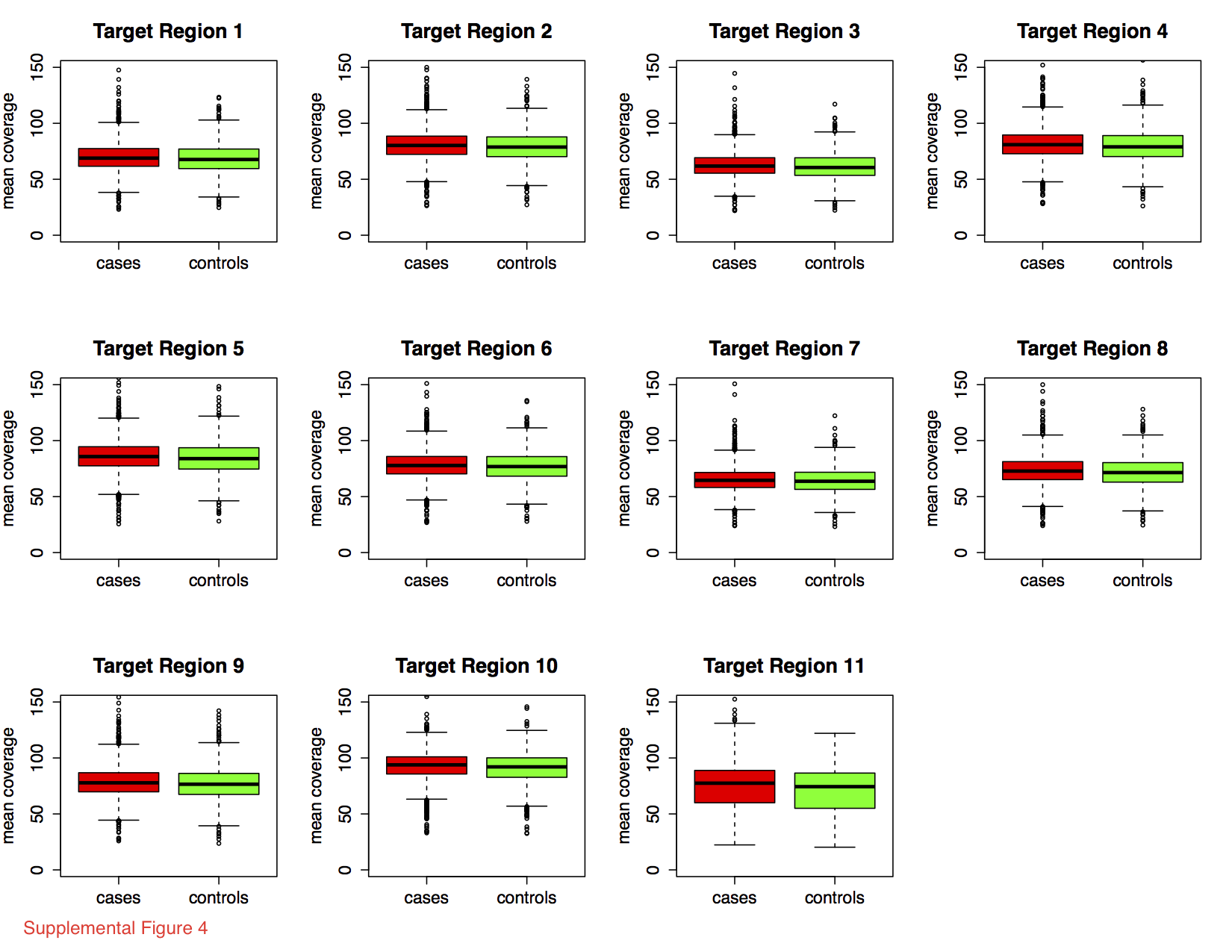

Supplement: Additional file 1: — Boxplot of per-sample coverage by region, for each of the sequenced regions, by case–control status. (TIFF 8219 kb) [file 12864_2016_2459_MOESM1_ESM.tiff]

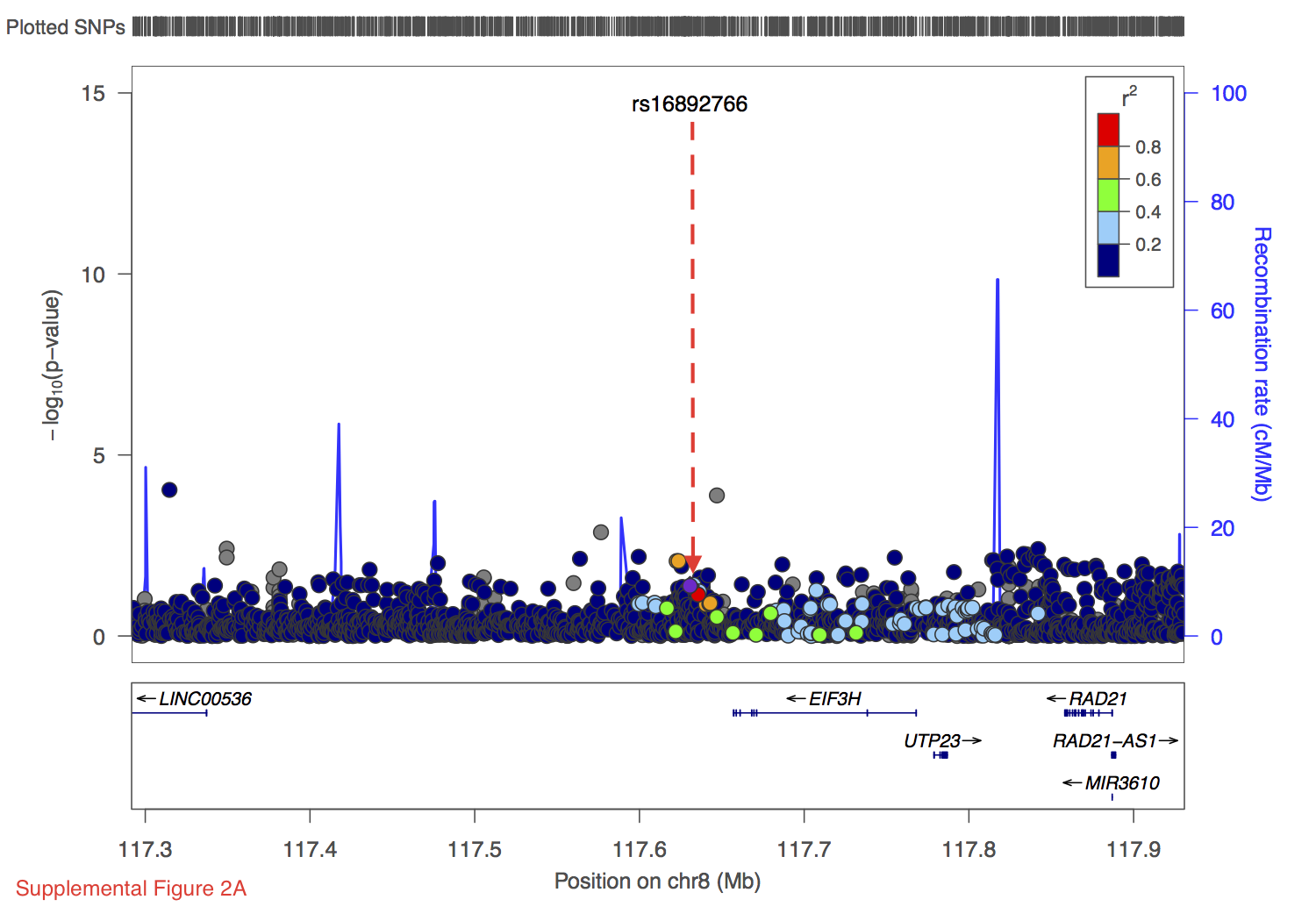

Supplement: Additional file 2: — Regional plots of associations for each targeted region. rs numbers and purple circles indicate the focal GWAS SNP that the region was selected around. Colored circles indicate degree of LD among SNPs. Grey circles indicate novel SNPs that lack LD information based on the 2012 release of the 1000 Genomes data. The rs number at figure top is centered around the location of the focal SNP. (ZIP 1921 kb) [file 12864_2016_2459_MOESM2_ESM.zip › suppl_figS2/suppl_figS2a.tiff]

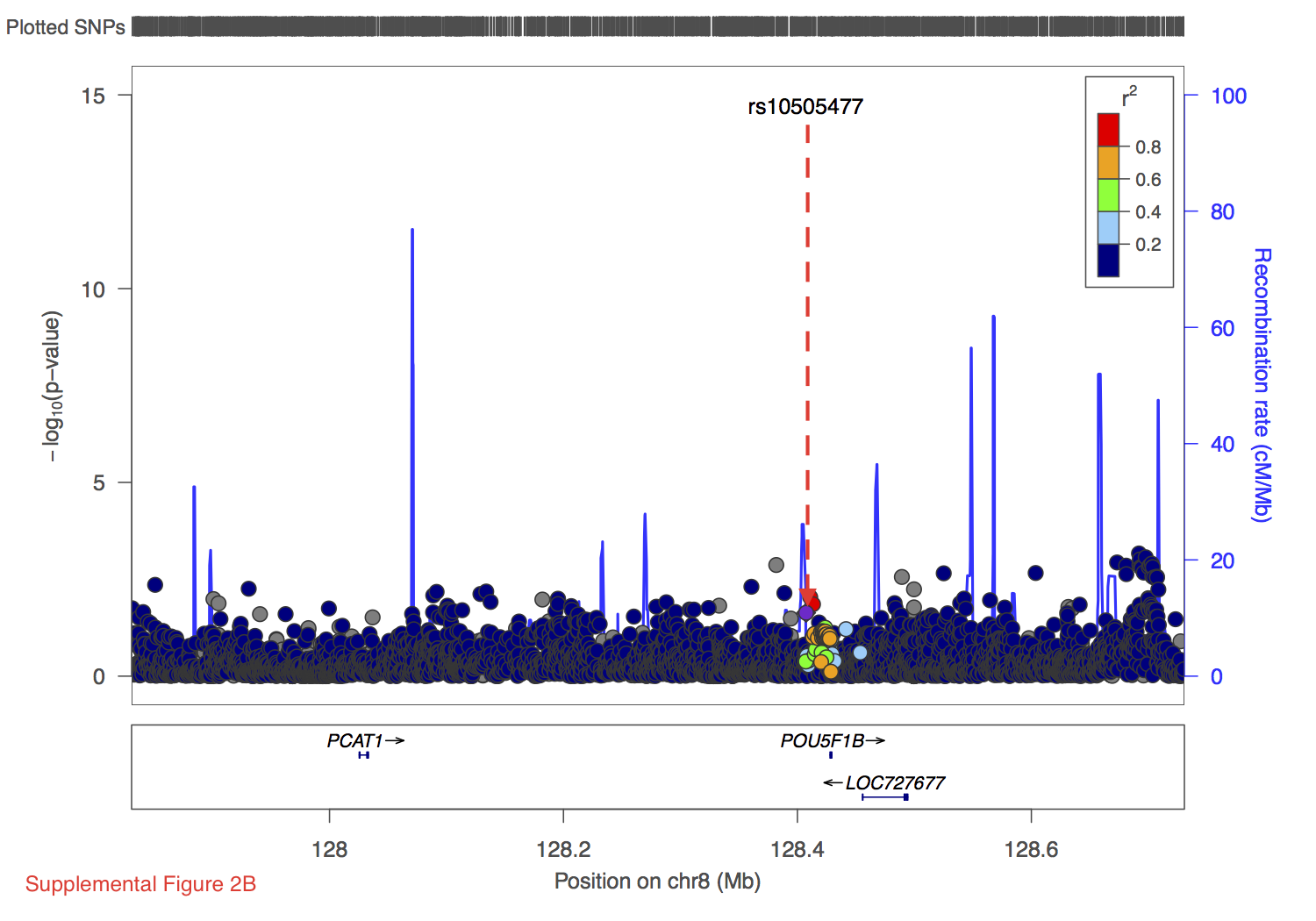

Supplement: Additional file 2: — Regional plots of associations for each targeted region. rs numbers and purple circles indicate the focal GWAS SNP that the region was selected around. Colored circles indicate degree of LD among SNPs. Grey circles indicate novel SNPs that lack LD information based on the 2012 release of the 1000 Genomes data. The rs number at figure top is centered around the location of the focal SNP. (ZIP 1921 kb) [file 12864_2016_2459_MOESM2_ESM.zip › suppl_figS2/suppl_figS2b.tiff]

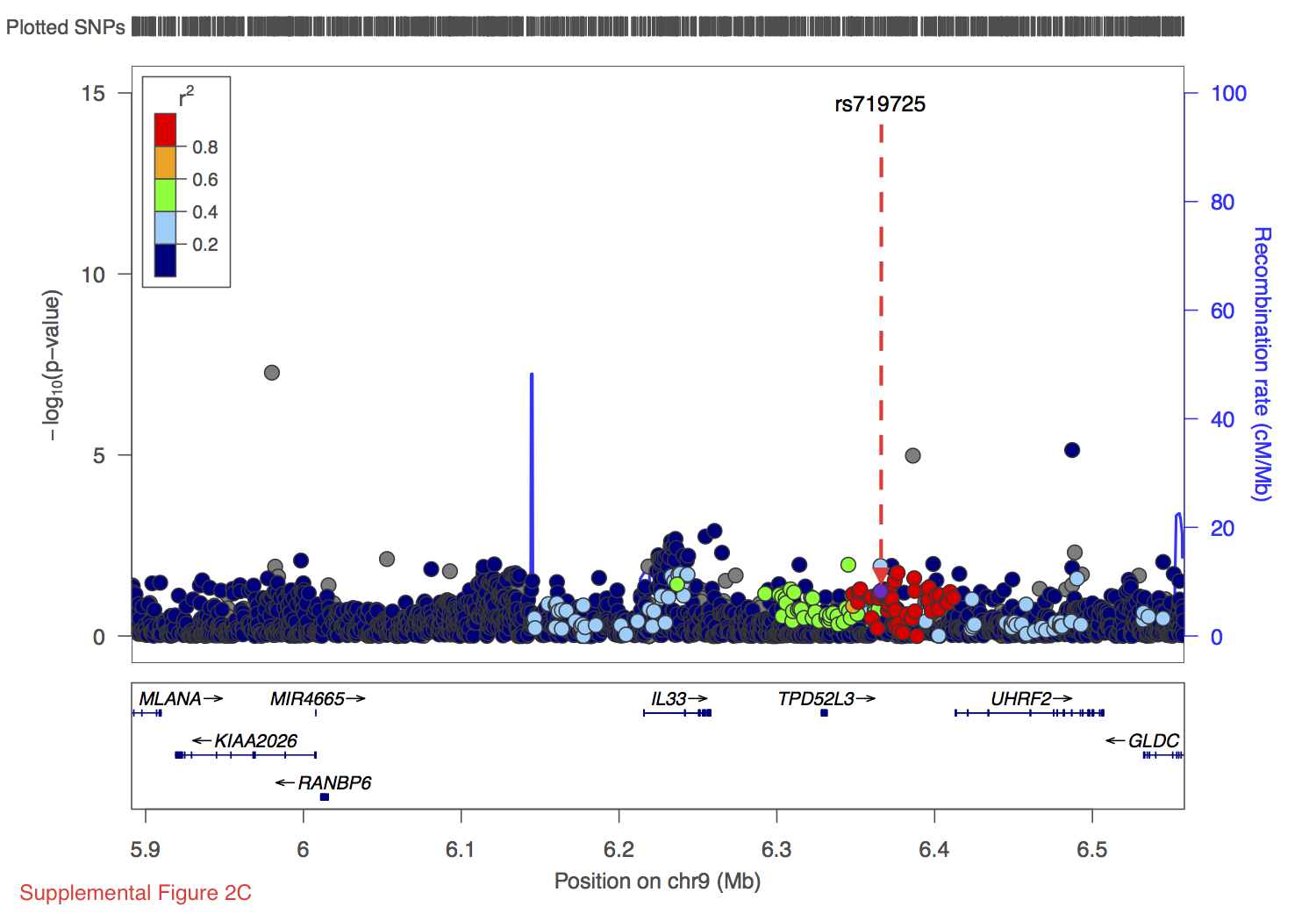

Supplement: Additional file 2: — Regional plots of associations for each targeted region. rs numbers and purple circles indicate the focal GWAS SNP that the region was selected around. Colored circles indicate degree of LD among SNPs. Grey circles indicate novel SNPs that lack LD information based on the 2012 release of the 1000 Genomes data. The rs number at figure top is centered around the location of the focal SNP. (ZIP 1921 kb) [file 12864_2016_2459_MOESM2_ESM.zip › suppl_figS2/suppl_figS2c.tiff]

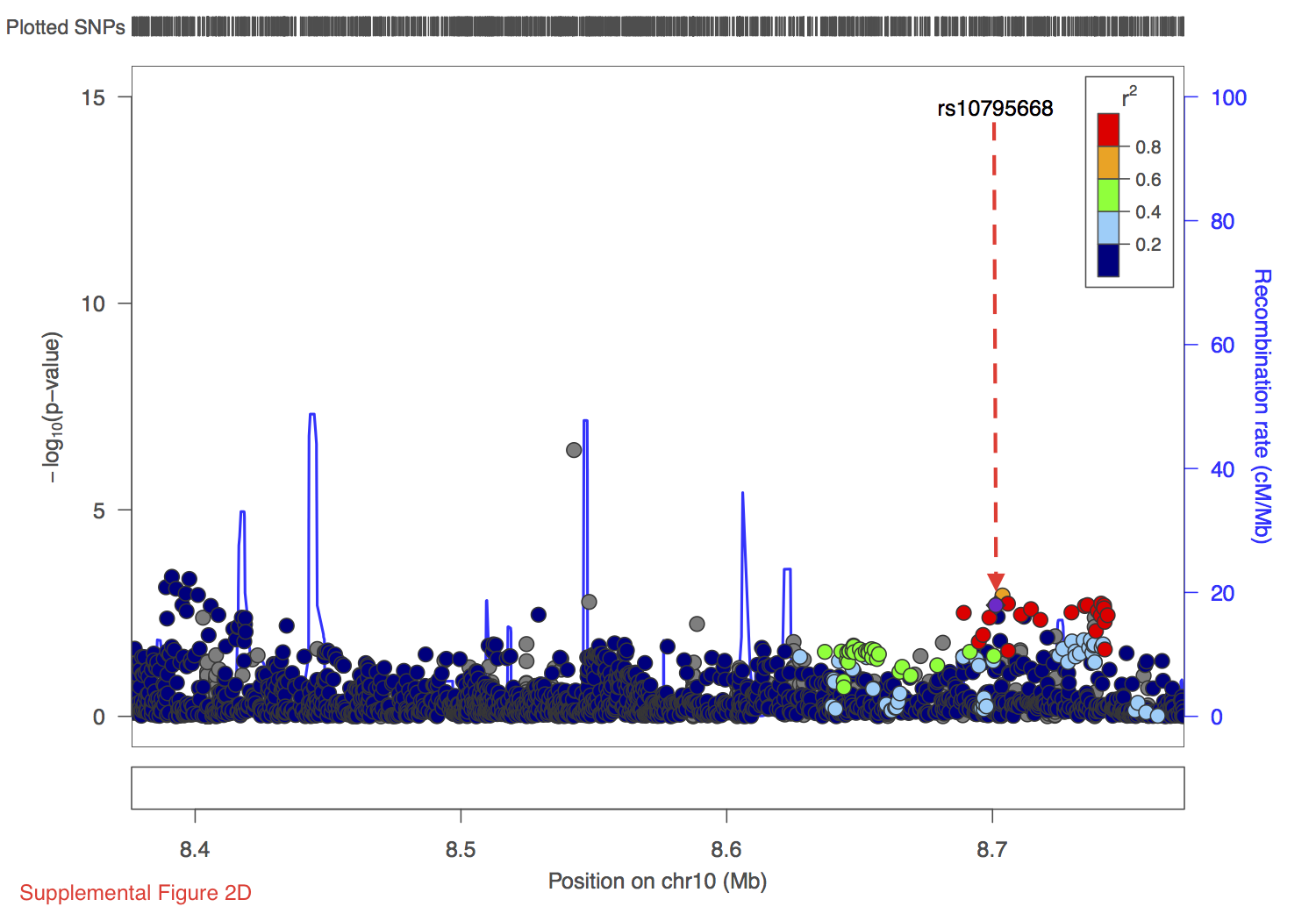

Supplement: Additional file 2: — Regional plots of associations for each targeted region. rs numbers and purple circles indicate the focal GWAS SNP that the region was selected around. Colored circles indicate degree of LD among SNPs. Grey circles indicate novel SNPs that lack LD information based on the 2012 release of the 1000 Genomes data. The rs number at figure top is centered around the location of the focal SNP. (ZIP 1921 kb) [file 12864_2016_2459_MOESM2_ESM.zip › suppl_figS2/suppl_figS2d.tiff]

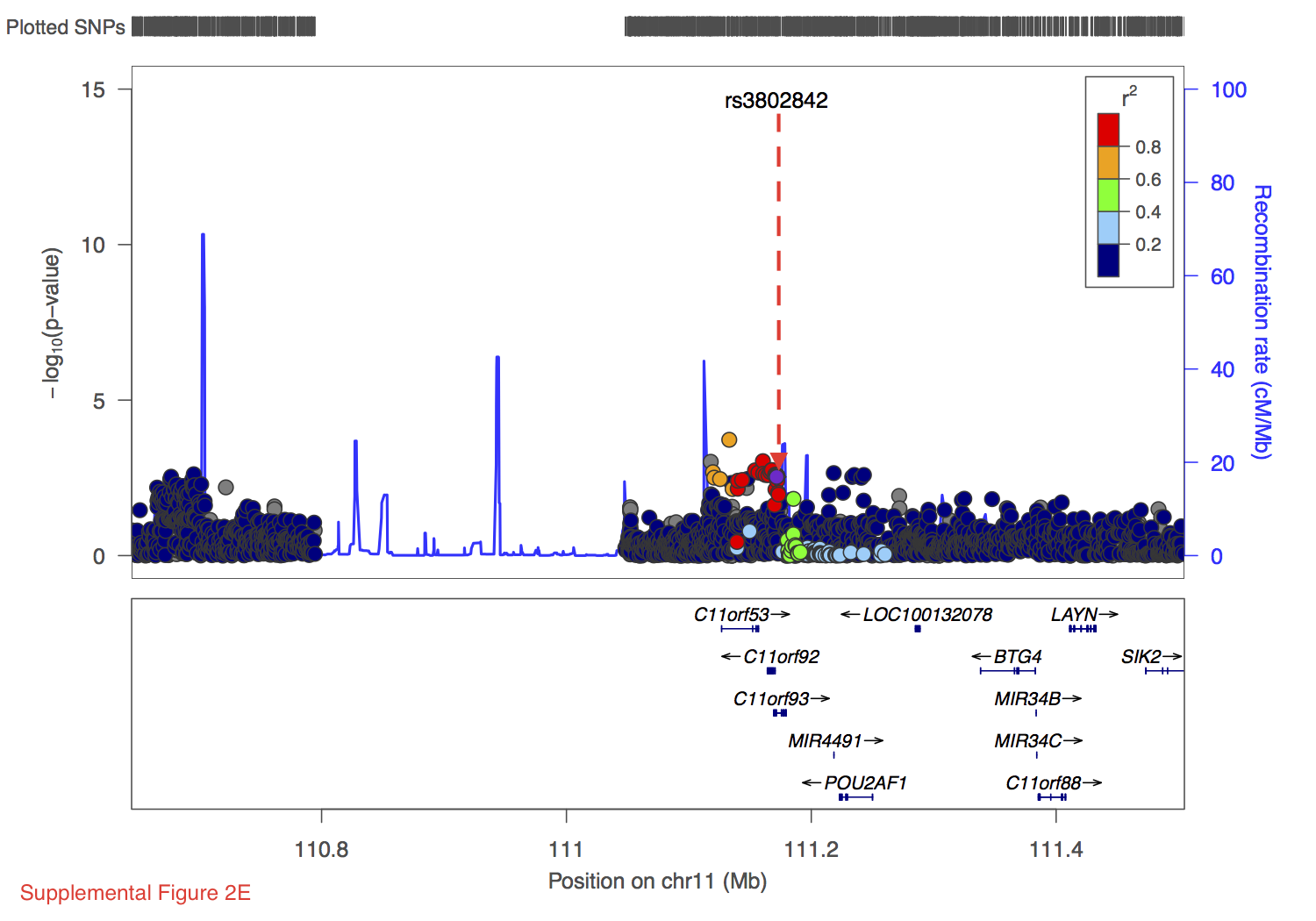

Supplement: Additional file 2: — Regional plots of associations for each targeted region. rs numbers and purple circles indicate the focal GWAS SNP that the region was selected around. Colored circles indicate degree of LD among SNPs. Grey circles indicate novel SNPs that lack LD information based on the 2012 release of the 1000 Genomes data. The rs number at figure top is centered around the location of the focal SNP. (ZIP 1921 kb) [file 12864_2016_2459_MOESM2_ESM.zip › suppl_figS2/suppl_figS2e.tiff]

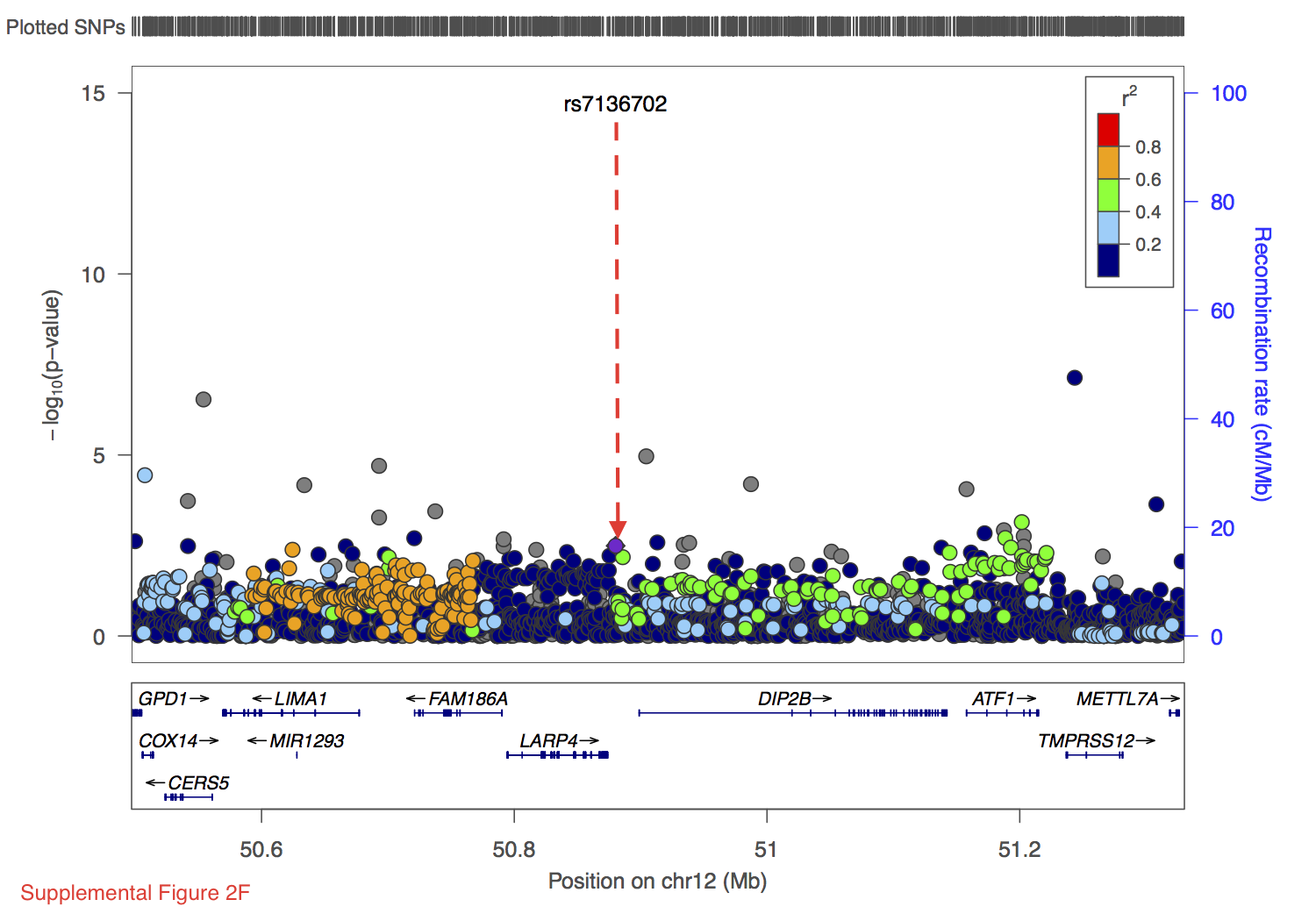

Supplement: Additional file 2: — Regional plots of associations for each targeted region. rs numbers and purple circles indicate the focal GWAS SNP that the region was selected around. Colored circles indicate degree of LD among SNPs. Grey circles indicate novel SNPs that lack LD information based on the 2012 release of the 1000 Genomes data. The rs number at figure top is centered around the location of the focal SNP. (ZIP 1921 kb) [file 12864_2016_2459_MOESM2_ESM.zip › suppl_figS2/suppl_figS2f.tiff]

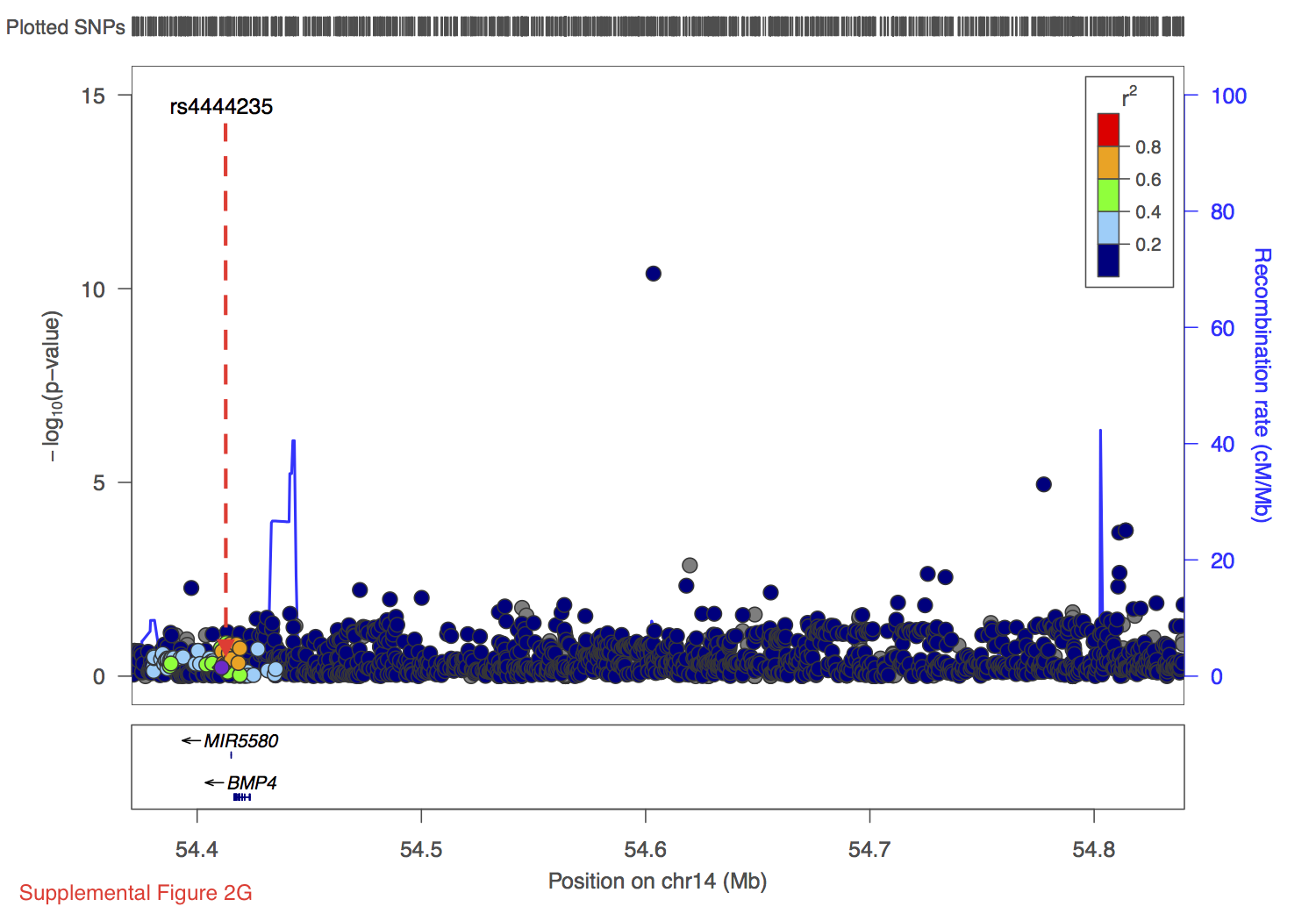

Supplement: Additional file 2: — Regional plots of associations for each targeted region. rs numbers and purple circles indicate the focal GWAS SNP that the region was selected around. Colored circles indicate degree of LD among SNPs. Grey circles indicate novel SNPs that lack LD information based on the 2012 release of the 1000 Genomes data. The rs number at figure top is centered around the location of the focal SNP. (ZIP 1921 kb) [file 12864_2016_2459_MOESM2_ESM.zip › suppl_figS2/suppl_figS2g.tiff]

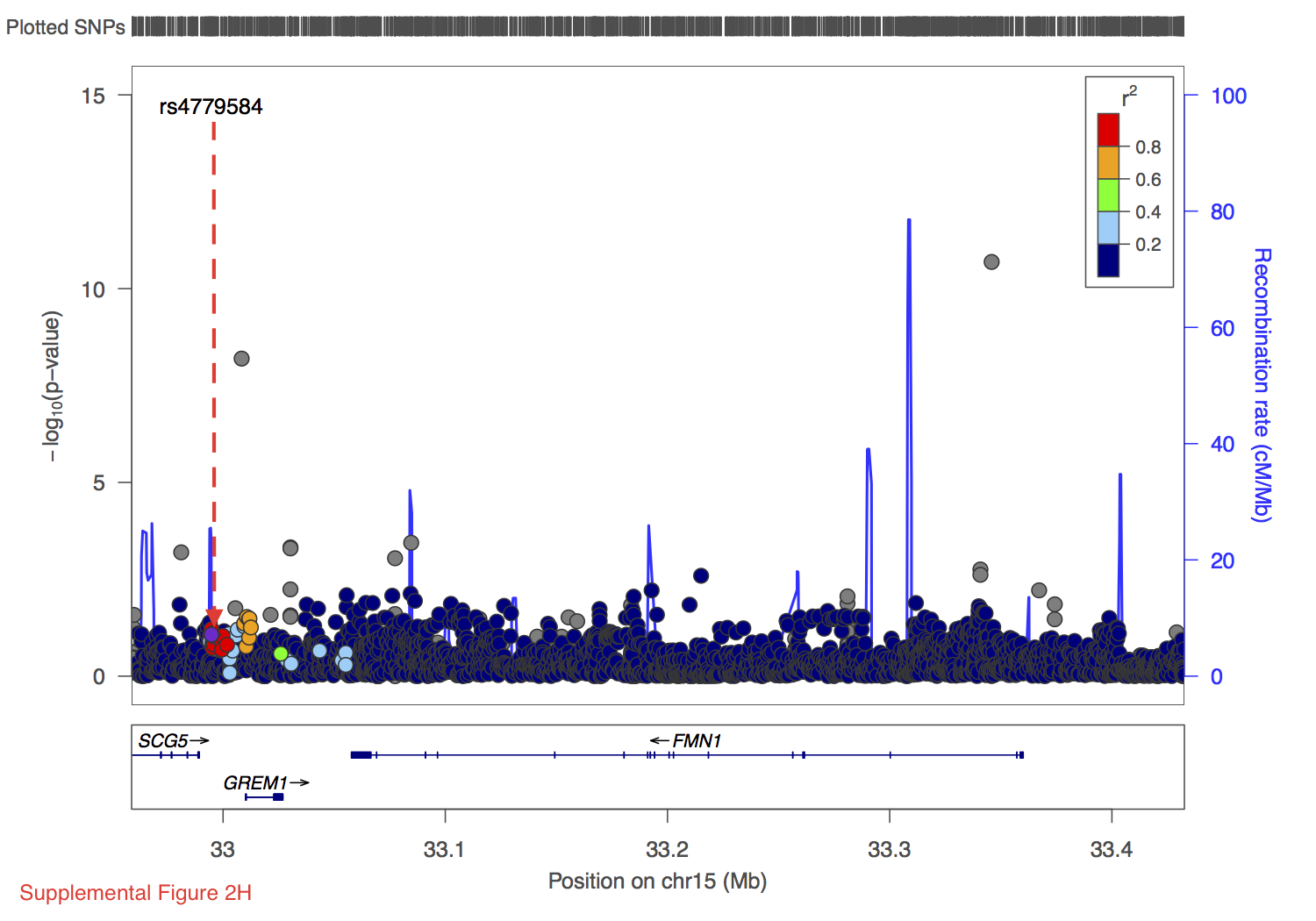

Supplement: Additional file 2: — Regional plots of associations for each targeted region. rs numbers and purple circles indicate the focal GWAS SNP that the region was selected around. Colored circles indicate degree of LD among SNPs. Grey circles indicate novel SNPs that lack LD information based on the 2012 release of the 1000 Genomes data. The rs number at figure top is centered around the location of the focal SNP. (ZIP 1921 kb) [file 12864_2016_2459_MOESM2_ESM.zip › suppl_figS2/suppl_figS2h.tiff]

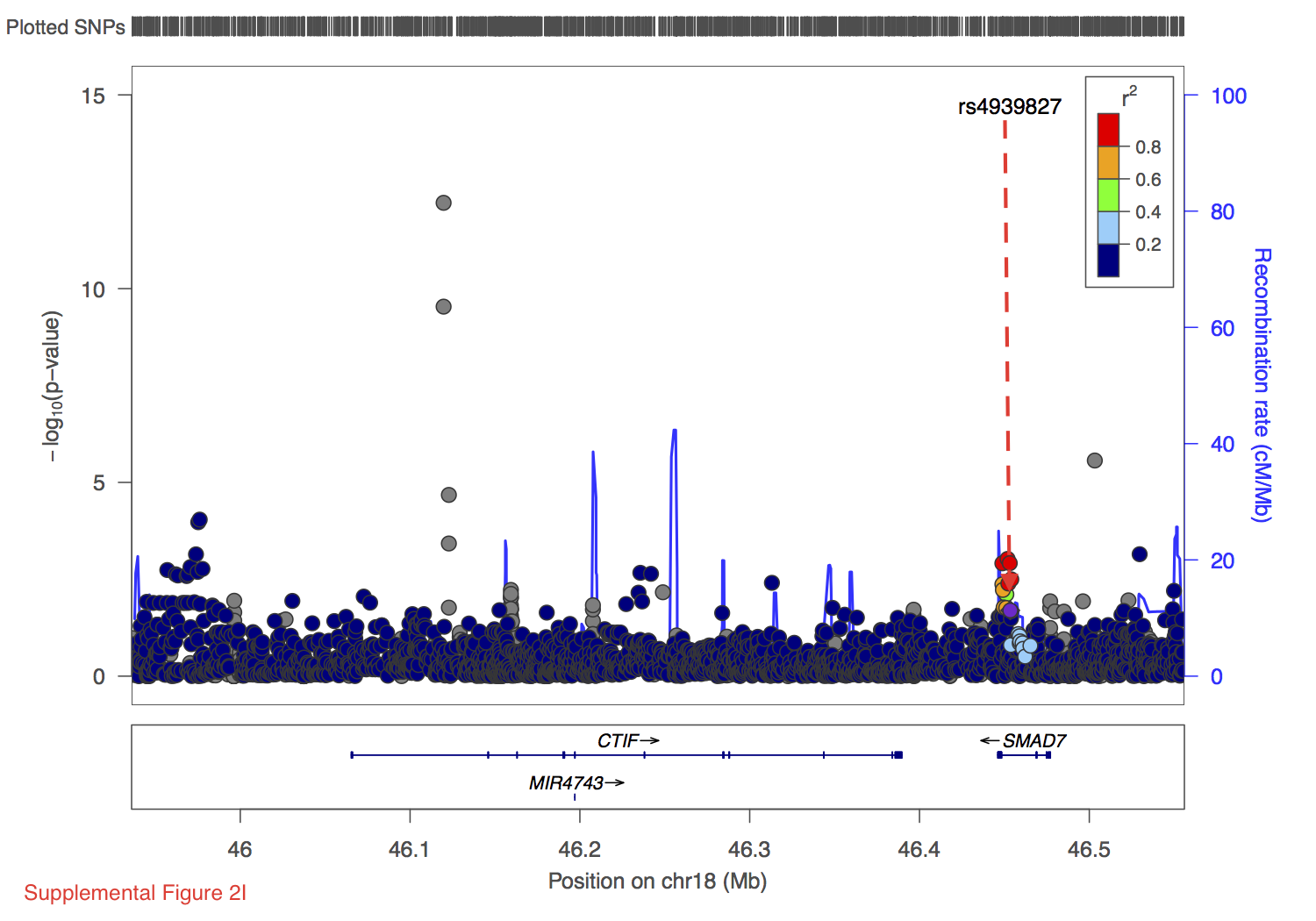

Supplement: Additional file 2: — Regional plots of associations for each targeted region. rs numbers and purple circles indicate the focal GWAS SNP that the region was selected around. Colored circles indicate degree of LD among SNPs. Grey circles indicate novel SNPs that lack LD information based on the 2012 release of the 1000 Genomes data. The rs number at figure top is centered around the location of the focal SNP. (ZIP 1921 kb) [file 12864_2016_2459_MOESM2_ESM.zip › suppl_figS2/suppl_figS2i.tiff]

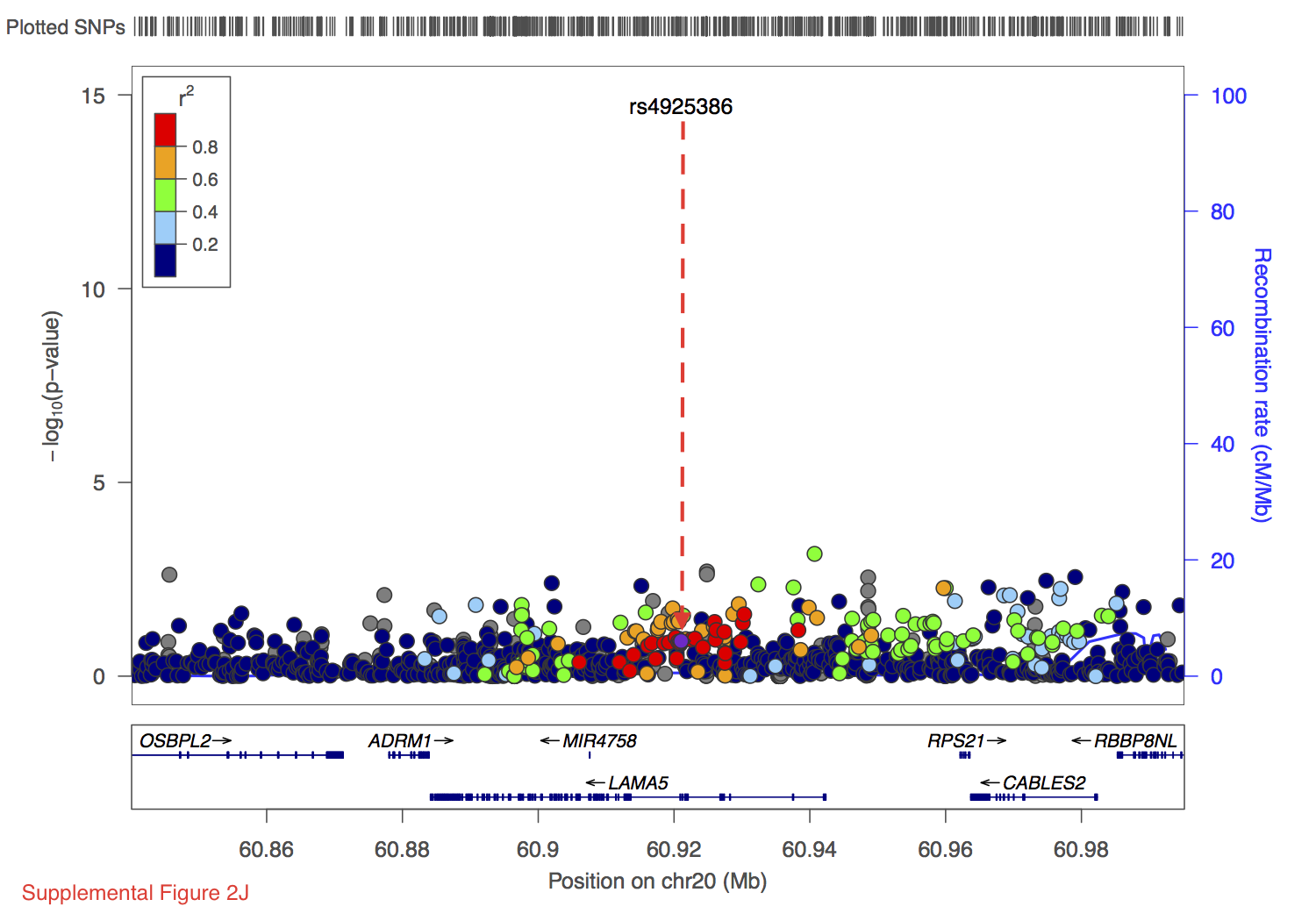

Supplement: Additional file 2: — Regional plots of associations for each targeted region. rs numbers and purple circles indicate the focal GWAS SNP that the region was selected around. Colored circles indicate degree of LD among SNPs. Grey circles indicate novel SNPs that lack LD information based on the 2012 release of the 1000 Genomes data. The rs number at figure top is centered around the location of the focal SNP. (ZIP 1921 kb) [file 12864_2016_2459_MOESM2_ESM.zip › suppl_figS2/suppl_figS2j.tiff]

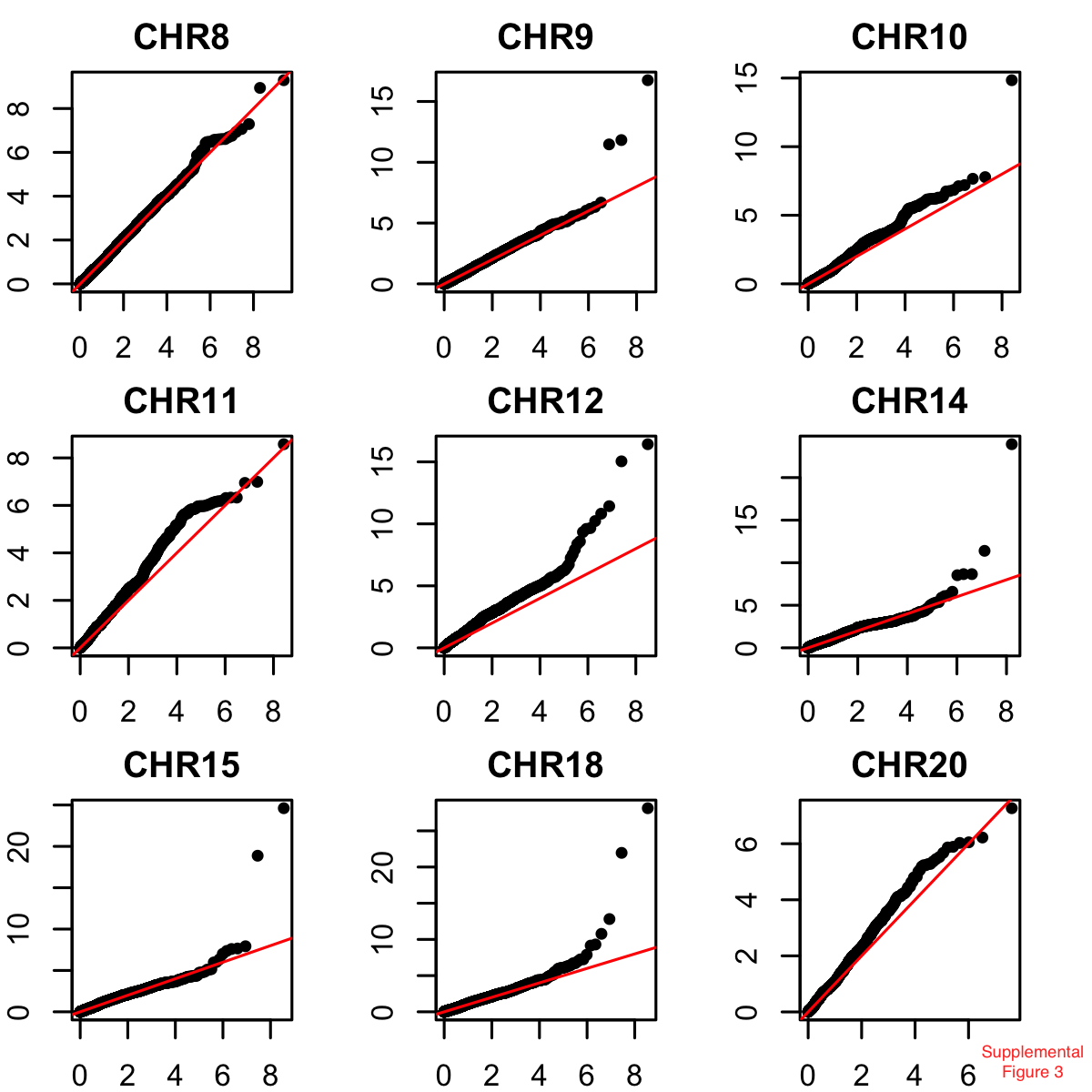

Supplement: Additional file 3: — ‘By region’ QQ-plot for marginal associations between common polymorphism and cancer status. X-axes show expected –log(p-value); y-axes shows observed –log(p-value). (TIFF 5627 kb) [file 12864_2016_2459_MOESM3_ESM.tiff]

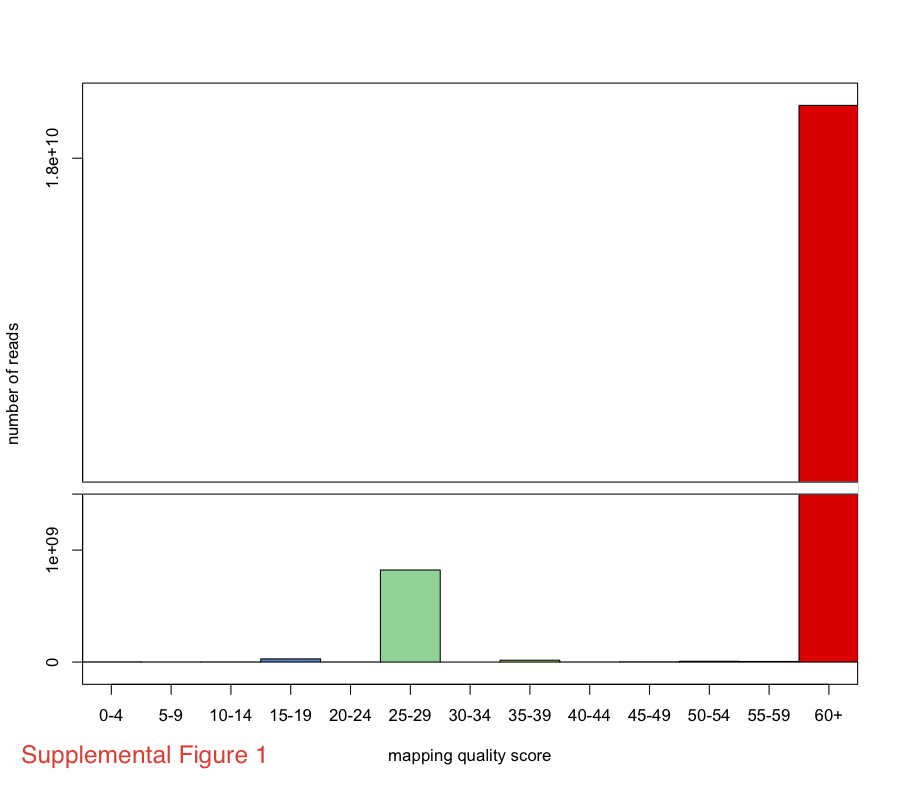

Supplement: Additional file 4: — Distribution of mapping qualities (MQ) across all samples. Reads with MQ < = 50 were excluded during variant calling to reduce false positive variant calls. (TIFF 2774 kb) [file 12864_2016_2459_MOESM4_ESM.tiff]
